# Supplementary figures and images for: Hydra: A mixture modeling framework for subtyping pediatric cancer cohorts using multimodal gene expression signatures
Source: PLoS Comput Biol. 2020 Apr 10;16(4):e1007753. doi: 10.1371/journal.pcbi.1007753 (PMC7176284; doi:10.1371/journal.pcbi.1007753)

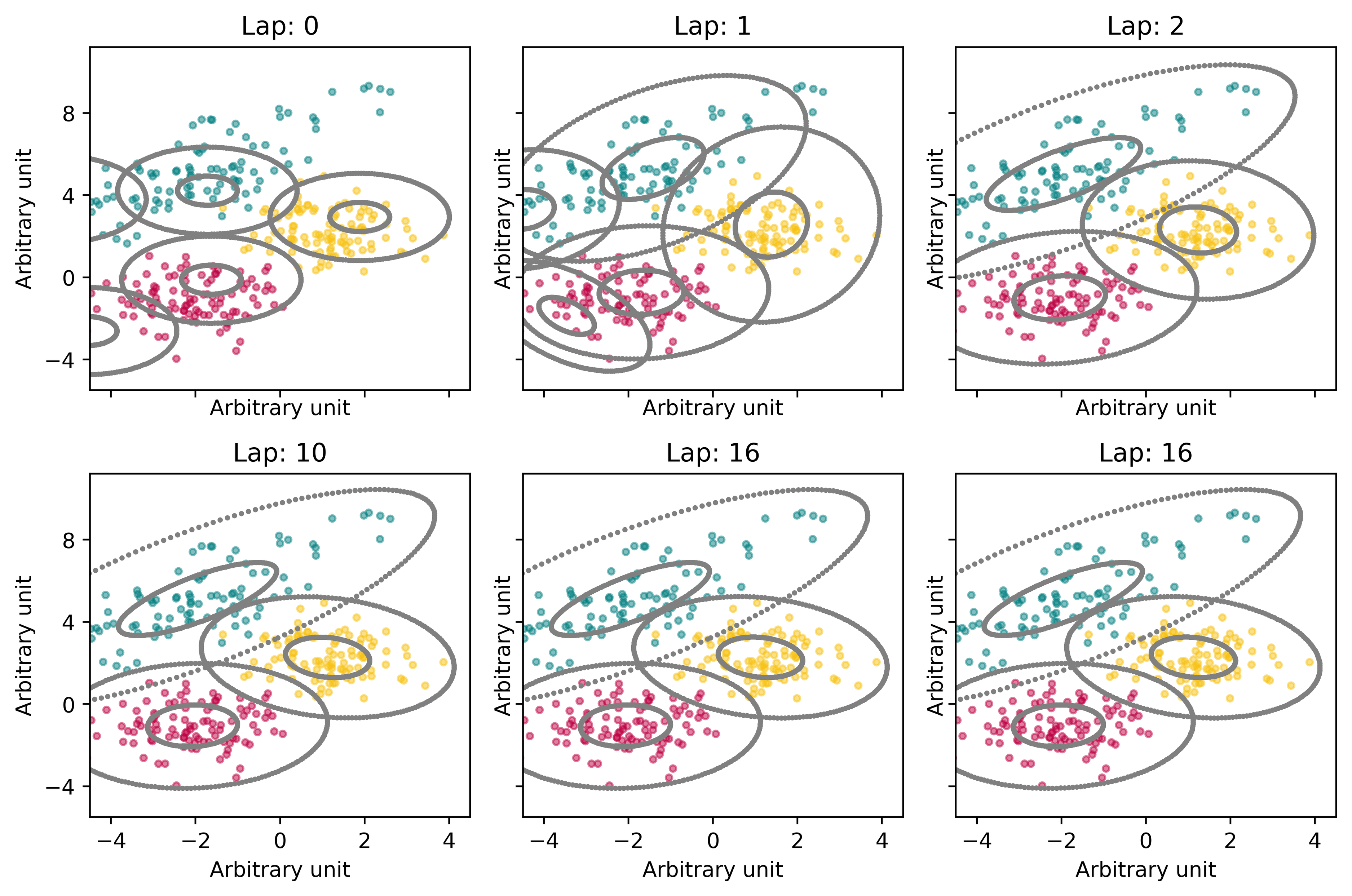

Supplement: S1 Fig — We used the bnpy moVB algorithm to infer the number of clusters from synthetic data. The model first randomly assigns clusters. Then, the model iteratively improves the model fit, creating and destroying clusters until the model converges on the correct number of clusters at lap 16 [56]. (TIF) [file pcbi.1007753.s001.tif]

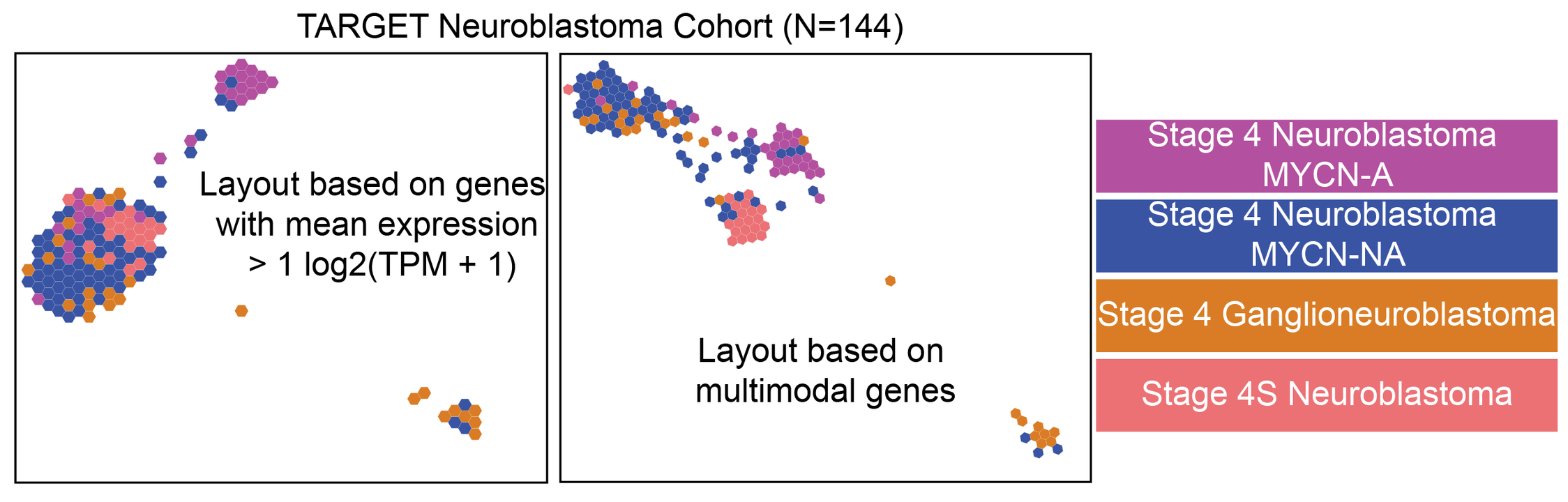

Supplement: S2 Fig — Standard TumorMap analysis of the TARGET neuroblastoma dataset resulted in stage 4S samples clustering with stage 4 neuroblastoma samples (left). An alternative TumorMap based solely on 1,498 multimodally expressed genes separated the stage 4S samples into a distinct cluster (right). (TIF) [file pcbi.1007753.s002.tif]

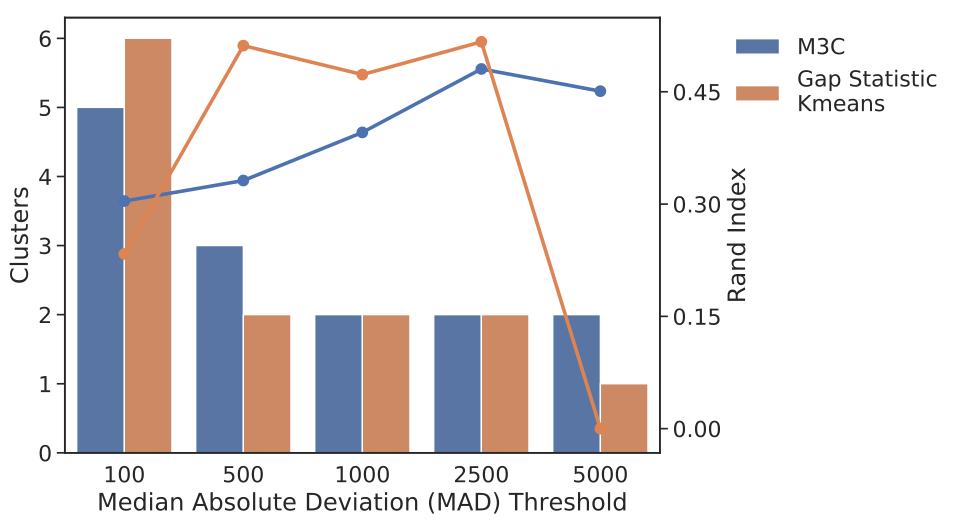

Supplement: S3 Fig — We tested a range of gene expression variation thresholds based on the median absolute deviation, but found that the clusters identified by this approach could not resolve the same clusters as the hydra approach. The barplot shows the number of clusters and the lineplot tracks the Rand index comparing the M3C and k-means clusters and the hydra clusters. (TIF) [file pcbi.1007753.s003.tif]

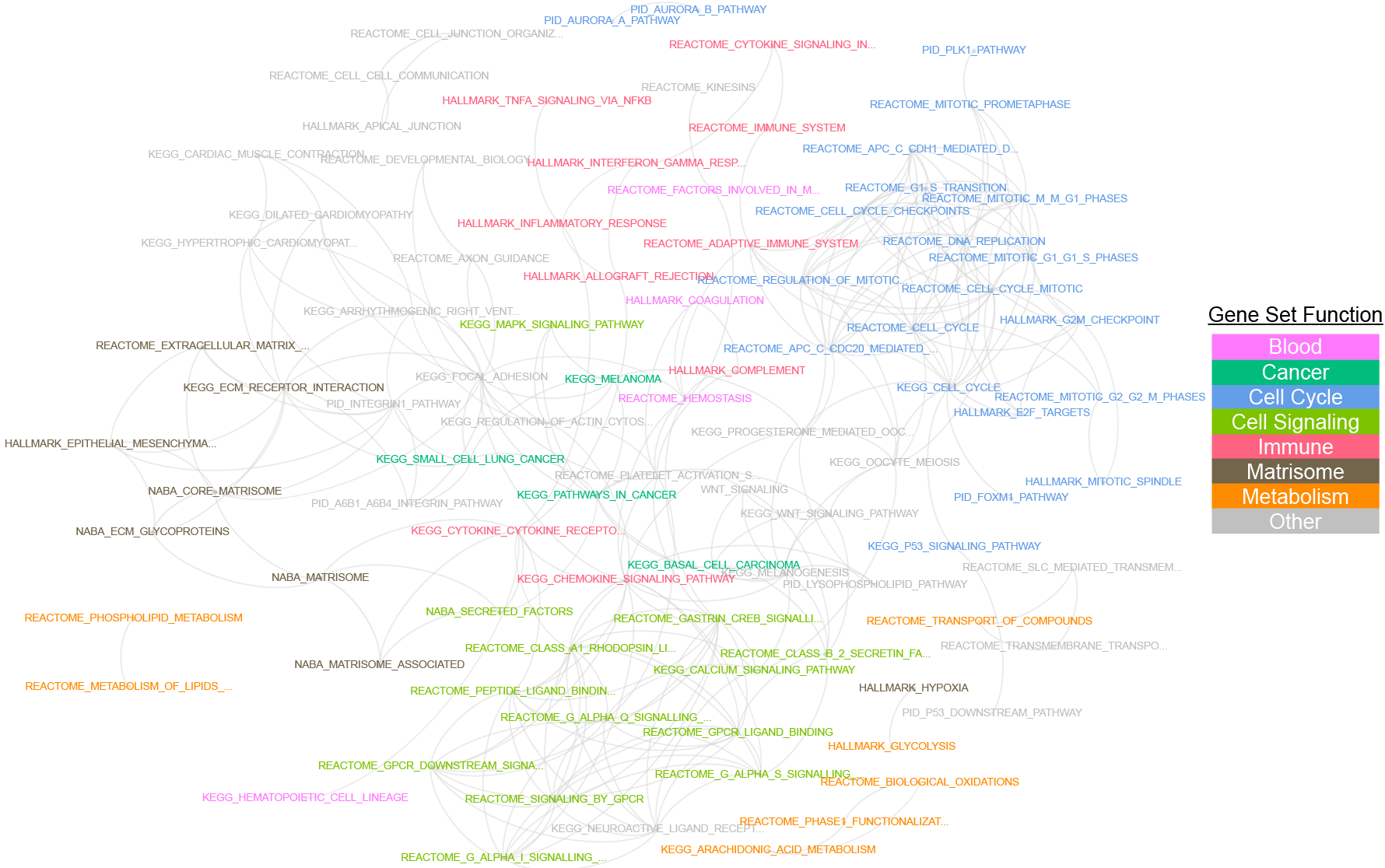

Supplement: S4 Fig — Unsupervised clustering of multimodal gene sets revealed biological themes associated with hallmark cancer functions, including cell cycle, immune cell signaling, extracellular matrix organization, and metabolism. (TIF) [file pcbi.1007753.s004.tif]

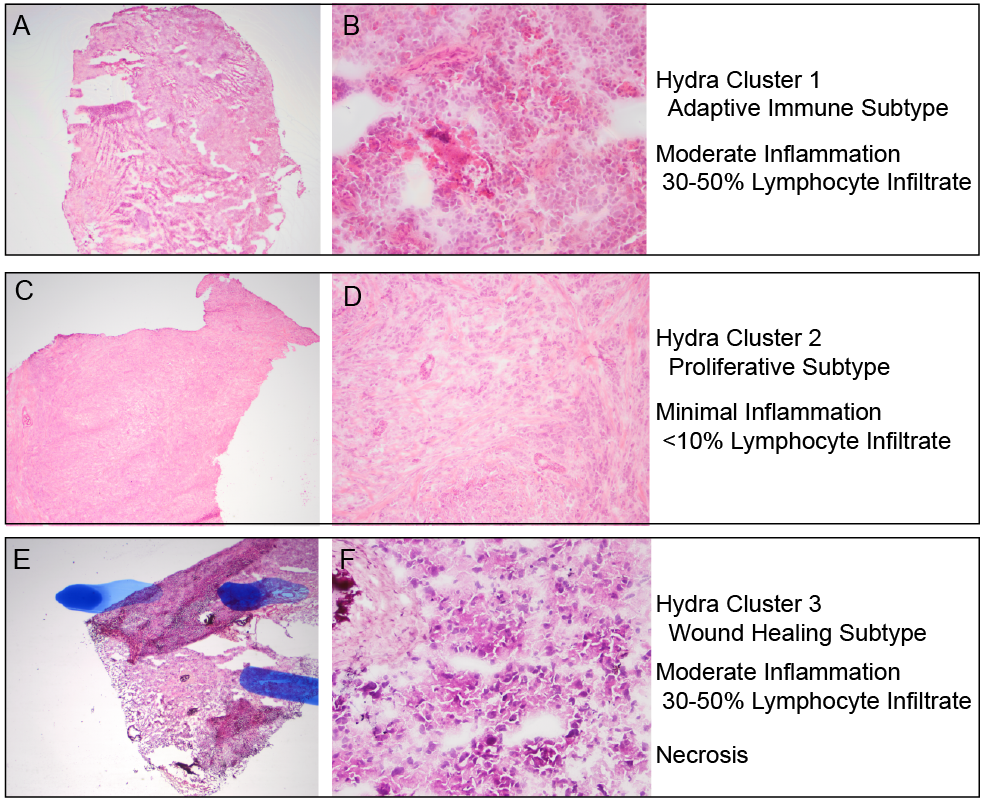

Supplement: S5 Fig — A-B: H&E sections from fresh frozen tumor tissue from MYCN-NA neuroblastoma sample at A: 2X magnification and B: 20X magnification. Tumor cells are medium to large with moderate amounts of cytoplasm and areas of rhabdoid appearing undifferentiated cells. There is a moderate amount of mixed inflammation present (30-50%) consisting mostly of mature mononuclear cells with some plasma cells and scattered eosinophils. C-D: H&E sections from fresh frozen tumor tissue from MYCN-NA neuroblastoma at C: 2X magnification and D: 20X magnification. Tumor cells are moderate to large in size with moderate amounts of cytoplasm. There is a minimal amount (<10%) of apparent mononuclear inflammation scattered throughout the tumor. E-F: H&E sections from fresh frozen tumor tissue from MYCN-NA neuroblastoma sample at (E) 2X magnification and (F) 20X magnification. Tumor cells are medium to large with moderate amounts of cytoplasm and areas of rhabdoid appearing undifferentiated cells. There are also areas of apparent necrosis. There is a moderate amount of inflammation present (30-50%) consisting mostly of mature mononuclear cells with some plasma cells and scattered eosinophils. (TIF) [file pcbi.1007753.s005.tif]

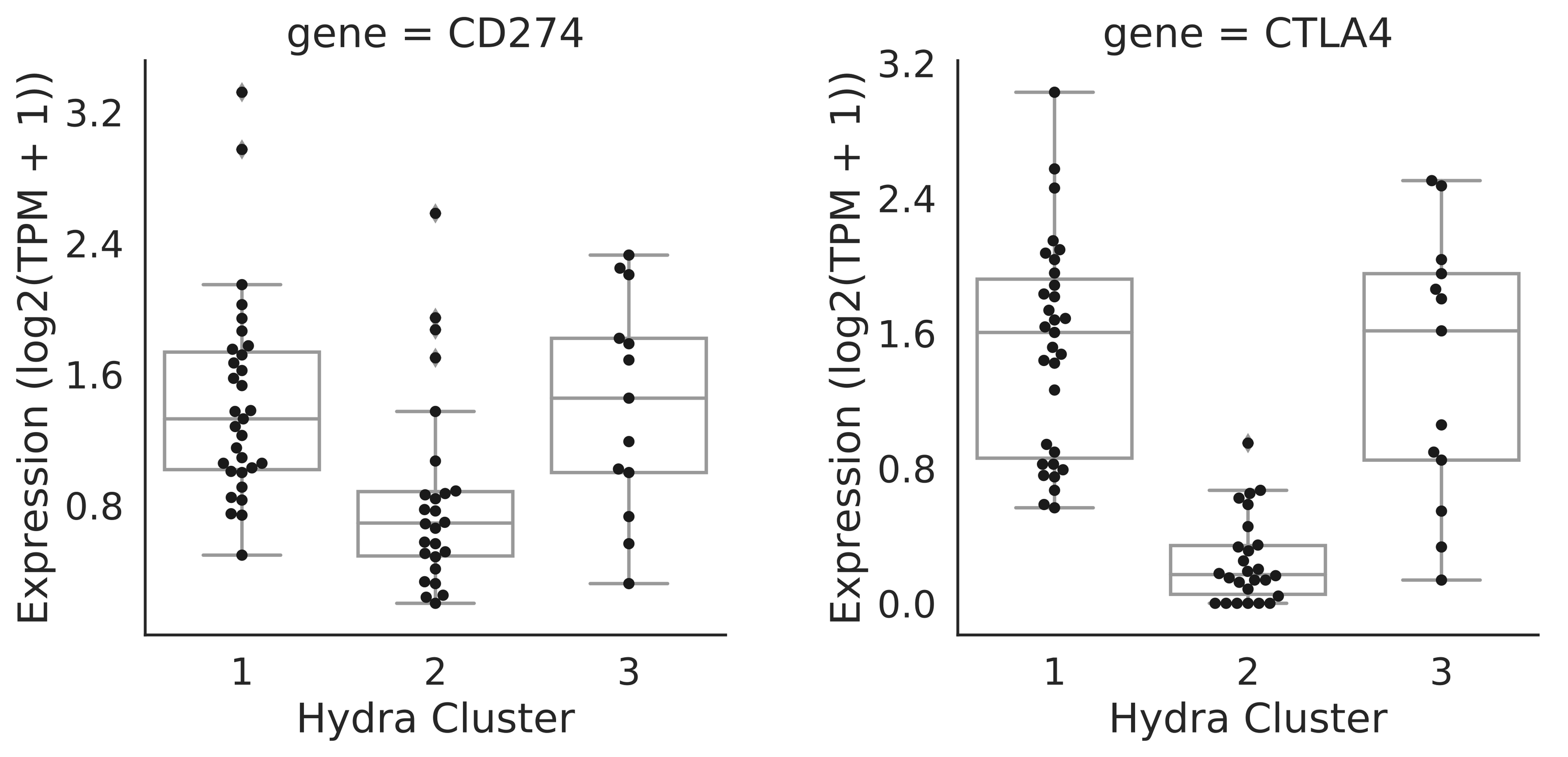

Supplement: S6 Fig — (TIF) [file pcbi.1007753.s006.tif]

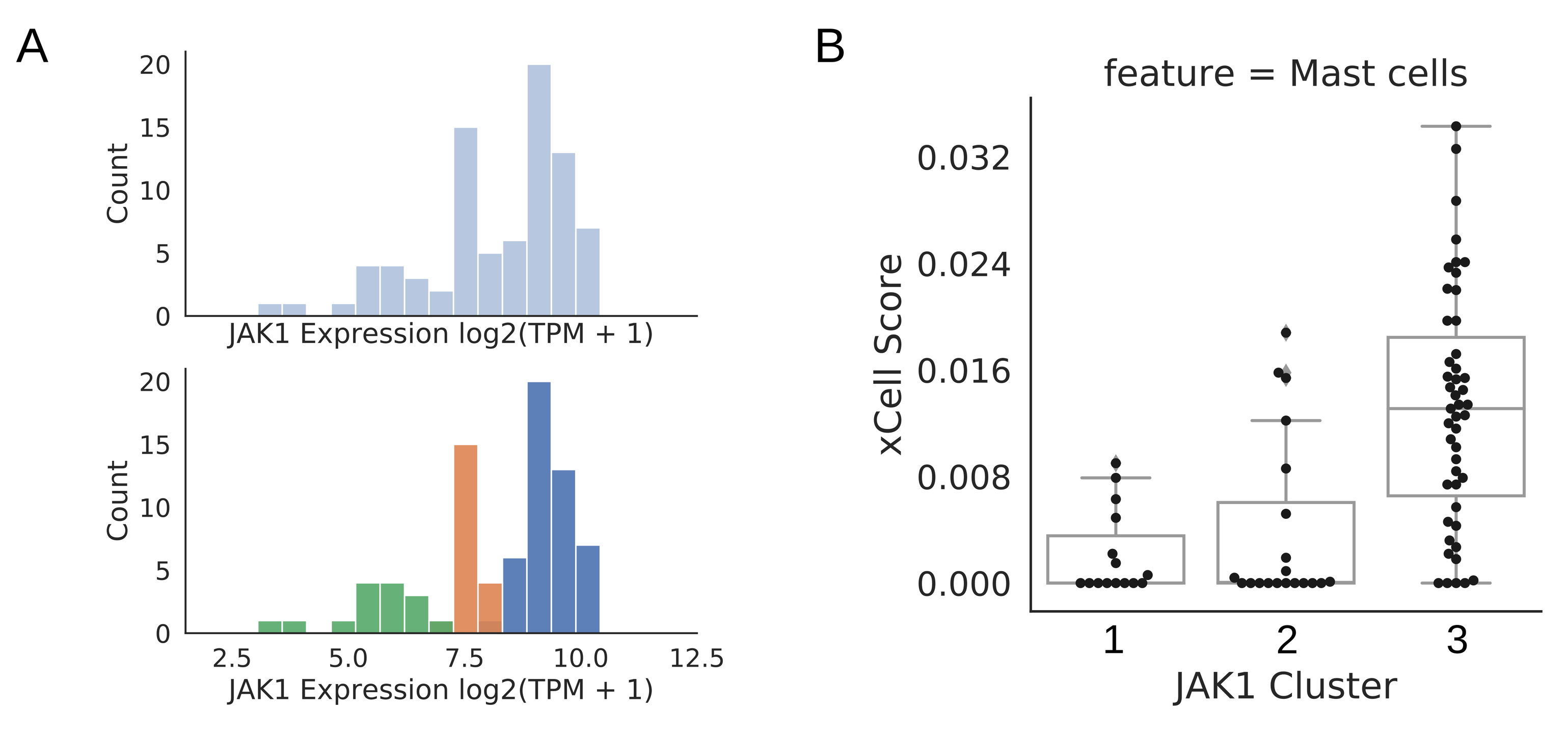

Supplement: S7 Fig — A: JAK1 expression distribution for Ewing sarcoma cohort (top) and the JAK1 expression distributions for cluster 1 (green), 2 (orange), and 3 (blue). B: Boxplot showing the xCell mast cell enrichment score for the three clusters associated with JAK1 expression. (TIF) [file pcbi.1007753.s007.tif]
