# Supplementary material for: Hydra: A mixture modeling framework for subtyping pediatric cancer cohorts using multimodal gene expression signatures
Source: PLoS Comput Biol. 2020 Apr 10;16(4):e1007753. doi: 10.1371/journal.pcbi.1007753 (PMC7176284; doi:10.1371/journal.pcbi.1007753)
Supplement: S2 File — (PDF) [file pcbi.1007753.s009.pdf]

Overview

There is a need for more flexible gene expression analyses for precision oncology applications. We developed an unsupervised clustering approach for identifying clinically relevant expression patterns. The hydra analysis framework uses state-of-the-art Bayesian non-parametric statistics to learn which genes are differentially expressed without the need for matched normal expression data. This is very useful in the pediatric oncology setting where matched normal tissue is usually not available.

The hydra pipeline includes routines for identifying multimodally distributed genes, scanning for differentially expressed gene sets, and identifying enriched gene sets from multimodally expressed genes. Hydra is available as a docker container for easy deployment (see figure below).

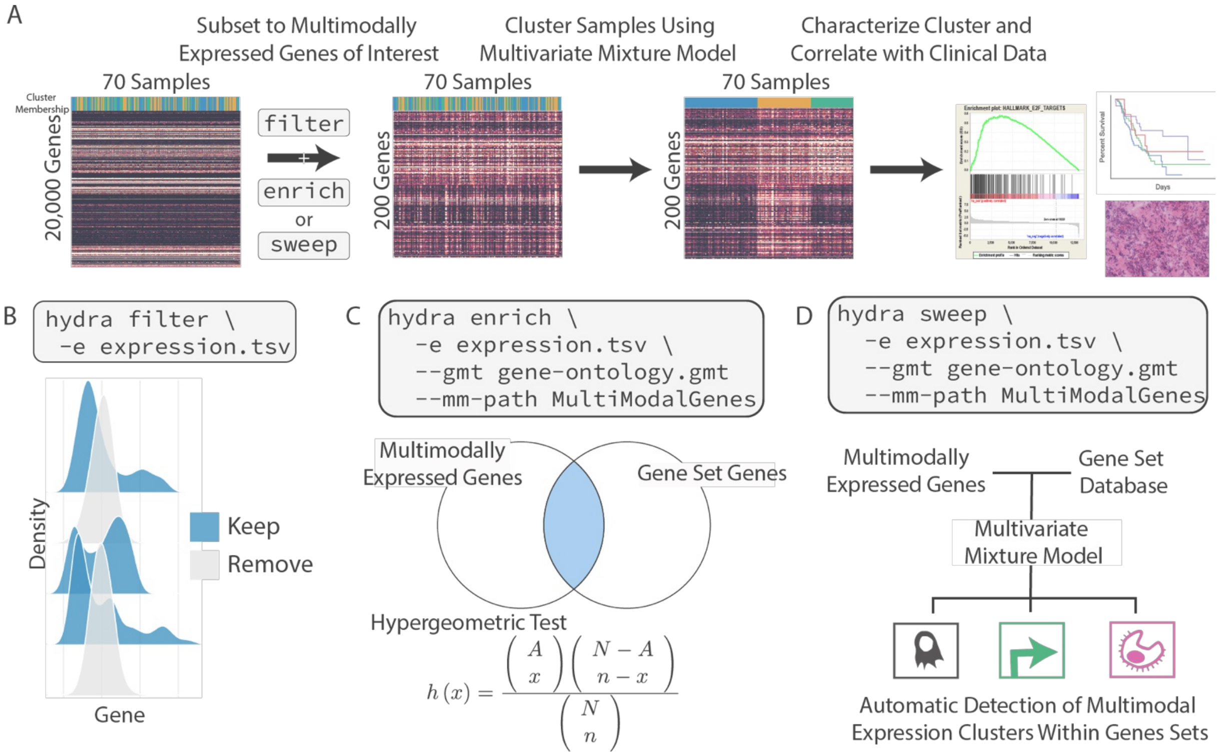

The first step in the hydra pipeline is the filter command, which identifies multimodally expressed (ME) genes in your expression dataset. After this, there are two main usage modes for hydra which utilize the ME gene list: supervised gene set analysis using the sweep command, and unsupervised gene-set clustering analysis using the enrich command. In both modes, a Jupyter notebook will be produced to analyze and visualize the results.

The *sweep* analysis is useful if you are interested in investigating gene sets or gene signatures that have known relevance in your disease of interest. The results of the sweep command can identify gene sets with the greatest power for differentiating subtypes of samples.

The *enrich* command is useful for generating new hypotheses about subtype-specific expression by identifying novel subtype clusters in your expression data through multivariate clustering with the ME gene list.

Options

Run the hydra command by itself or with -h flag to see all options and descriptions of all arguments:

```
docker run -it -v $PWD:/data jpfeil/hydra:0.2.4 -h
```

Test:
Test data is available in the hydra/test directory along with a bash script with example commands.

Step 1: Identify Multimodally Expressed Genes Using filter

Use the filter tool to identify multimodally expressed genes in your expression dataset.

Flags:
`-e [path to expression TSV file; samples as columns and genes as rows]`
`-o [path to output directory]`

```
docker run -it -v $PWD:/data jpfeil/hydra:0.2.4 filter \
-e test/test-exp.tsv \
-o test-filter \
--CPU 15
```

This will generate a MultiModalGenes directory. The next step in the pipeline is to perform supervised or unsupervised clustering analysis with sweep or enrich.

Step 2: Identify coordinated expression of multimodally expressed genes

Option 1: Supervised Gene Set Analysis Using Hydra *sweep*

The *sweep* command is designed to search for differentially expressed gene sets in a supervised manner, given a gene expression dataset and a GMT file of gene set annotations. (Make sure that the GMT file gene annotations match the annotations of the gene expression matrix; for example, ENSEMBL or Hugo gene annotations.) Gene sets can be found in the hydra github repository under the gene-sets directory.

Flags:
`-e [path to expression TSV file; samples as columns and genes as rows]`
`-o [path to output directory]`
`--min-mean-filter [minimum expression mean for gene]`
`--gmt [path to GMT file]`
`--gmt-regex [Regex for subsetting gmt file to specific gene sets]`

```
docker run -it -v $PWD:/data jpfeil/hydra:0.2.4 sweep \
-e test/test-exp.tsv \
-o test-sweep \
--min-mean-filter 1.0 \
--gmt /opt/hydra/gene-sets/h.all.v6.2.symbols.gmt \
--gmt-regex IL2_STAT5
--CPU 15
```

After running the *sweep* analysis you can use the jupyter notebook to analyze the results (see last section of

this README)).

~

Option 2: Unsupervised Enrichment Analysis Using *enrich*
The *enrich* command finds enrichment of multimodally expressed genes within a user-defined database of gene sets. There are two ways to perform the *enrich* analysis. The first way is to use the command-line tool, but we actually recommend using the Jupyter notebook approach because it provides more flexibility for investigating clusters. We will first present the command-line approach, but we encourage the user to also read the Jupyter notebook approach below.

The *enrich* method includes an important parameter known as the minimum component probability. This is an additional filter to remove multimodally expressed genes that influence a small subset of your samples. This parameter gives you the ability to subset the enriched genes to those that influence a greater number of patients. Before running *enrich*, you can use the *scan* command in the jupyter notebook to adjust the minimum component probability during multivariate clustering.

Perform GO enrichment clustering across multimodally expressed genes

```
docker run -it -v $PWD:/data jpfeil/hydra:0.2.4 enrich \
-e <PATH to expression tsv file> \
-m <PATH to MultiModalGenes dir> \
--min-prob-filter 0.1 \
--go-enrichment \
-o <output directory>
```

Or you can perform enrichment analysis using a user-specified gene set with the --gmt flag. The enrichment analysis uses the clusterProfiler tool, which requires the gene set database use entrez ids. The gene expression input matrix should still use gene symbols.

```
docker run -it -v $PWD:/data jpfeil/hydra:0.2.4 enrich \
-e test/test-exp.tsv \
-m <PATH to MultiModalGenes dir> \
--min-prob-filter 0.1 \
--gmt /opt/hydra/gene-sets/h.all.v6.2.entrez.gmt \
-o test-enrich
```

Now you can use the jupyter notebook to analyze the results (see next section).

Step 3: Analyze results with Jupyter notebook
Interactive environment for investigating expression data. This comes with all of the hydra code and dependencies pre-installed.

```
docker run -it -v $(pwd):/data/ -p 8889:8888 jpfeil/hydra:0.2.4 notebook -e None
```

The token for accessing the Jupyter notebook is printed to stdout.

Direct your browser to the following link:

http://127.0.0.1:8889

Input the token given at stdout.

The first step is to add the hydra library to your path from within the docker container.

```
import sys
sys.path.append('/opt/hydra/')
import library.analysis as hydra
```

If working with sweep results, you can use this code snippet to identify which gene-sets are “hits”.

```
hits = hydra.SweepAnalysis().rank(<path to MultivariateAnalysis directory>)
```

This provides the number of clusters identified and the Kullback–Leibler divergence, which is a measure of how different the clusters are in expression space. We recommend prioritizing gene-sets with a large Kullback-Leibler divergence, to identify clusters that have significantly different expression patterns.

It is also possible to perform the *enrich* analysis in a Jupyter notebook on your laptop. All you need is the path to the input expression matrix and the directory of MultiModalGene models.

The minimum component probability filter can be used to tune the resolution of the clustering analysis with respect to the number of samples available. We provide a method ScanEnrichmentAnalysis to explore how the minimum probability thresholds influence gene set enrichment and the number of clusters.

```
scan = hydra.ScanEnrichmentAnalysis(mm_path,
                                    exp_path,
                                    'GO',
                                    CPU=7,
                                    cluster=True).scan()
```

Then in the EnrichmentAnalysis method, use a min_prob_filter from the ScanEnrichmentAnalysis results which maximizes the number of clusters.

```
mm = hydra.EnrichmentAnalysis(mm_path, # PATH to MultiModelGenes dir
                              exp_path, # PATH to gene expression input
                              min_prob_filter=0.2,
                              gmt_path='GO')

terms = mm.get_enriched_terms()
genes = mm.get_enriched_term_genes()

clus = hydra.MultivariateMixtureModel(data=exp.reindex(genes),
                                      center=True,
                                      gamma=5.0,
                                      variance=2.0,
                                      K=5)

assignments = clus.get_assignments(exp.reindex(genes))
```

We also provide routines for characterizing clusters using GSEA.

```
features = clus.get_cluster_features(exp,  # Original expression DataFrame
                                     gmt='/opt/hydra/gene-sets/h.all.v6.2.symbol.gmt')
```

This analysis provides the gene-sets that are enriched in each cluster. This can be used to identify biological themes within the cluster, including the tumor microenvironment state and druggable pathway expression. Our analysis of gene expression data found that tumor microenvironment signals dominate, so to subtract this signal and identify potential underlying expression signatures, we implementes the sub_cluster_gsea routine.

```
subclust = clus.sub_cluster_gsea(sample_exp,
                                 gmt='/opt/hydra/gene-sets/h.all.v6.2.symbol.gmt')
```
